# Uric acid causes kidney injury through inducing fibroblast expansion, Endothelin-1 expression, and inflammation

**DOI:** 10.1186/s12882-017-0736-x

**Published:** 2017-10-31

**Authors:** Muhammad Mansyur Romi, Nur Arfian, Untung Tranggono, Wiwit Ananda Wahyu Setyaningsih, Dwi Cahyani Ratna Sari

**Affiliations:** 1 Department of Anatomy, Faculty of Medicine, Universitas Gadjah Mada, Yogyakarta, Indonesia; 2 Department of Surgery, Faculty of Medicine, Universitas Gadjah Mada, Yogyakarta, Indonesia

**Keywords:** Hyperuricemia, ET-1, Glomerulosclerosis, Tubular injury, Fibroblast expansion, Inflammation

## Abstract

**Background:**

Uric acid (UA) plays important roles in inducing renal inflammation, intra-renal vasoconstriction and renal damage. Endothelin-1 (ET-1) is a well-known profibrotic factor in the kidney and is associated with fibroblast expansion. We examined the role of hyperuricemia conditions in causing elevation of ET-1 expression and kidney injury.

**Methods:**

Hyperuricemia was induced in mice using daily intraperitoneal injection of uric acid 125 mg/Kg body weight. An NaCl injection was used in control mice. Mice were euthanized on days-7 (UA7) and 14 (UA14). We also added allopurinol groups (UAL7 and UAL14) with supplementation of allopurinol 50 mg/Kg body weight orally. Uric acid and creatinine serum were measured from blood serum. Periodic Acid Schiff (PAS) and Sirius Red staining were done for glomerulosclerosis, tubular injury and fibrosis quantification. mRNA expression examination was performed for nephrin, podocin, preproEndothelin-1 (ppET-1), MCP-1 and ICAM-1. PDGFRβ immunostaining was done for quantification of fibroblast, while α-SMA immunostaining was done for localizing myofibroblast. Western blot analysis was conducted to quantify TGF-β1, α-SMA and Endothelin A Receptor (ETAR) protein expression.

**Results:**

Uric acid and creatinine levels were elevated after 7 and 14 days and followed by significant increase of glomerulosclerosis and tubular injury score in the uric acid group (*p* < 0.05 vs. control). Both UA7 and UA14 groups had higher fibrosis, tubular injury and glomerulosclerosis with significant increase of fibroblast cell number compared with control. RT-PCR revealed down-regulation of nephrin and podocin expression (*p* < 0.05 vs. control), and up-regulation of MCP-1, ET-1 and ICAM-1 expression (*p* < 0.05 vs. control). Western blot revealed higher expression of TGF-β1 and α-SMA protein expression. Determination of allopurinol attenuated kidney injury was based on reduction of fibroblast cell number, inflammation mediators and ppET-1 expression with reduction of TGF-β1 and α-SMA protein expression.

**Conclusions:**

UA induced glomerulosclerosis, tubular injury and renal fibrosis with reduction of podocyte function and inflammatory mediator elevation. ET-1 and fibroblast expansion might modulate hyperuricemia induced renal fibrosis.

## Background

Chronic Kidney Diseases (CKDs) are one ofthe major global health problems in elderly populations due to their increased incidence and prevalence involving aging and declining health [1]. Recently, it was thought that an elevated uric acid level is common in these conditions and it increases the risk factors associated with heart disease, such as obesity, insulin resistance and gout. Some studies suggested that an elevated uric acid level may not only be a true risk factor but also may be one of the most important risk factors for cardiovascular disease. In renal diseases, the mechanism of hyperuricemia inducing the progression of renal failure is still not well understood. However, uric acid has been reported to play a role in renal inflammation, intra-renal vasoconstriction and renal failure induced by urate crystal formation [2, 3]. In cardiovascular diseases, uric acid may induce endothelial dysfunction with reduction of vasodilatation function [3].

Urate is a result of purine metabolism. In most species, this is an intermediate product which is degraded further by the hepatic enzyme uricase to allantoin, which then is excreted freely in the urine. Humans do not have uricase, due to a genetic mutation resulting in a non-functional form of the gene [4]. As a consequence, humans have higher serum urate levels (>2 mg/dl) compared with most mammals (<2 mg/dl). While hyperuricemia is defined as7 mg/dl in men and 6 mg/dl in women [5], uric acid levels also vary significantly within humans as the result of factors which increase the generation (such as high purine or protein diets, alcohol consumption, conditions with high cell turnover, or enzymatic defects in purine metabolism) or decreased excretion. A reduction in glomerular filtration rate (GFR) increases serum uric acid, although a significant compensatory increase in gastrointestinal excretion occurs. Hyperuricemia also may result from increased net tubular absorption. After filtration, uric acid undergoes both reabsorption and secretion in the proximal tubule, and this process is mediated by a urate/anion exchanger and a voltage-sensitive urate channel [6].

An increasing number of experimental and epidemiological studies suggest that uric acid is an independent risk factor for cardiovascular and renal disease [7, 8]. Uric acid can predict a high mortality in patients with hypertension, heart failure and diabetes [7, 9]. In renal diseases, hyperuricemia has already been known to contribute to the CKDs progression and is known as a marker of renal diseases [10]. In the 1950s-1980s the association between elevated serum uric acid and hypertension was observed and reported, although it still received less attention due to lack of a mechanistic explanation [11, 12]. Twenty-five to 40% of adult patients with untreated hypertension have hyperuricemia (>6.5 mg/dl), and this number increases dramatically when serum uric acid in the high-normal range is included [13]. One study reported that high uric acid levels were present in 25% of untreated hypertensive subjects, in 50% of subjects taking diuretics, and in 75% of subjects with malignant hypertension. The increase in serum uric acid in hypertension may be due to the decrease in renal blood flow which accompanies the hypertensive state, since a low renal blood flow will stimulate urate reabsorption [5].

The mechanisms of organ injury induced by uric acid remain incompletely understood, although growing evidences suggest that uric acid is an agent of inflammation [7, 14]. Raising levels of uric acid also had been reported to occur in rats with administration of uricase inhibitor and led to thickening of afferent arteriole, activation of the renin-angiotensin system, and hypertension [15, 16]. Uric acid also stimulates rat vascular smooth muscle cell (VSMC) proliferation in-vitro with increased expression of platelet-derived growth factor (PDGF), cyclooxygenase-2 (COX-2), and monocyte chemoattractant protein1 (MCP-1) [17, 18]. The autocrine or paracrine ET-1 system has already been known to be involved in the pathogenesis of CKDs for at least two decades. Endothelin-1 (ET-1) is a potent vasoconstrictor for the first time observed by Yanagisawa [19]. Here we demonstrated that hyperuricemia conditions induced renal damage with tubular injury and kidney fibrosis through activation of Endothelin-1 (ET-1) and fibroblast expansion.

## Methods

### Animal experiment: the hyperuricemia model

Hyperuricemia condition was induced in Swiss-background mice(4 months old, 30-35 g, *n* = 6-7 each group). Uric acid 125 mg/kg body weight (Sigma, U25G-26) was injected intraperitoneally for 7 (UA7 group) and 14 days (UA14 group). We also added an allopurinol group with supplementation of allopurinol 50 mg/kg body weight (Sigma, A8003) orally for 7 (UAL7 group) and 14 days (UAL14 group). The control group received NaCl 0.9% injection for 14 days. Animals were treated properly, using approved procedures in accordance with the guidelines of the Animal Care and Use Method of Faculty of Medicine, Universitas Gadjah Mada, Yogyakarta, Indonesia.

### Blood sampling and organs harvesting

According to the research schedule before euthanizing, blood was collected from the retro-orbital venous sinus using hematocrit capillary tubes. Kidneys were harvested, and the left kidney was used for RNA and protein examination and kept in RNAlater (Ambion, AM7021) solution before extraction. The right kidney was fixed in 4% PFA and embedded in paraffin for histo-staining examination. Creatinine and uric acid level were measured from the serum.

### Histological analysis and IHC staining

Four-mm paraffin sections were deparaffinized, stained for Periodic Acid Schiff to evaluate tubular injury and Sirius Red to quantify kidney fibrosis. Immunohistochemical (IHC) staining was done for these following antibodies after heated in Citrate Buffer and inhibited for endogenous peroxide using 3% H_2_O_2_ in PBS: Platelet-Derived Growth Factor Receptor β (PDGFRβ, Abcam ab32570, 200× dilution) and alpha-Smooth Muscle Actin (α-SMA, Sigma A2547, 400× dilution). Antibodies were incubated overnight. Secondary antibodies incubations were done for 1 h using appropriate secondary antibodies.

### Tubular injury and glomerulosclerosis score

Tubular injury and glomerulosclerosis were scored based on PAS staining*.* For tubular injury, scoring was done by grading tubular injury, epithelial cell apoptosis, intra-luminal cast and brush border loss in 15 randomly chosen, non-overlapping fields (100× magnification). Glomerulosclerosis was scored based on histopathological finding of glomerulosclerosis in 20 randomly non-overlapped glomerulus (400× magnification). The lesions were graded on a scale from 0 to 4, i.e.: 0: normal; 1: the injury involved less than 25% of the field (for tubular injury) and glomerulus; 2: the injury involve 25 to 50%; 3: the injury involved 50 to 75%; and 4: extensive injury involving more than 75% [20]. Kidney fibrosis area fraction was quantified using ImageJ software in 15 randomly chosen, non-overlapping fields (400× magnification) for each sample.

#### RNA extraction, real time and semi-quantitative reverse transcriptase PCR assay

Total RNA was extracted from kidney tissues using RNAiso PLUS (Takara Bio, Tokyo, Japan). RNA (1 μg) was reverse transcribed using ReverTra Ace Reverse Transcriptase (TOYOBO Co.,TRT-101) in a 20 μL reaction having random primer. Cycling conditions were 30 °C for 10 min, 42 °C for 60 min, and 99 °C for 5 min. Complementary DNA was diluted 15 times, then used for RT-PCR. Real-time PCR (qRT-PCR) was performed to examine ppET-1 expression using Kappa SYBR Fast Master Mix 2× (KAPA Biosystem, KK4600) using Biorad qRT-PCR Machine (Biorad, CFX96) with duplicates for each sample. Reverse Transcription PCR (RT-PCR) was performed to examine expressions of Nephrin, Podocin, preproET-1 (ppET-1/ET-1 mRNA), Monocyte Chemoattractant Protein-1 (MCP-1), and Intercellular Adhesion Molecule-1 (ICAM-1). The level of ppET-1 expression was quantified to determine ET-1 level. The reaction mixture (1 μL) was then used as the template in a conventional PCR assay. GoTag Green Master Mix (Promega, M7122) was used. The initial denaturation was performed at temperature 94 °C for 2 min. The condition for 30 cycles PCR were 94 °C for 10 s, 60 °C for 20 s, 72 °C for 1 min, and the last extension was 72 °C for 10 min. These following primer sets were used in this experiment: Nephrin: CCCAGGTACACAGAGCACAA (forward) and CTCACGCTCACAACCTTCAG (reverse), Podocin: GTGTCCAAAGCCATCCAGTT (forward) and GCAATGCTCTTCCTTTCCAG (reverse); ppET-1: CTGTGCACGCACCAGAGATG (forward) and AAGCATCAGTTGTGGCCTGTTAGA (reverse), Monocyte Chemoattractant Protein-1 (MCP-1): CTACAGACAACCACCTCAAGCACTTCTGTAG (forward) and GGCATCACAGTCCGAGTCACAC (reverse), Inter Cellular Adhesion Molecule-1 (ICAM-1): CAATTCACACTGAATGCCAGCTC (forward) and CAAGCAGTCCGTCTCGTCCA (reverse); and GAPDH: TTGCTGTTGAAGTCGCAGGAG (forward) and TGTGTCCGTCGTGGATCTGA (reverse). GAPDH expression was used for endogenous control. The PCR products were subjected to 2% agarose (Agarose S; Nippon Gene, Tokyo, Japan) gel electrophoresis and gel red staining (Bioron, Germany, Cat. No. 306009). The expression of PCR product in gel electrophoresis was quantified using densitometry analyses by imageJ software.

### Protein extraction and western blot

Protein was extracted using the Pro-Prep™ (Intron Biotechnology; Cat. No. 17081) from kidney tissue based on manufacturer instructions. Thirty milligrams of kidney tissues were homogenized with 600 μL of Pro-Prep™ solution. The homogenates were centrifuged at 12,000 rpm at 4 °C for 20 min. The supernatants were stored in safe lock tubes at -80 °C until they were assayed. A total of 40 μg of protein was separated onto 10% SDS-PAGE, and transferred to a polyvinylidene fluoride membrane (PVDF) and incubated with anti-Transforming Growth Factor-β1 (anti-rabbit TGF-β1, Cell Signaling 3711, 1:500 dilution), Anti-Endothelin A Receptor (anti-rabbit, ETAR, Santa Cruz sc-33,535, 1:300) and alpha-Smooth Muscle Actin (anti-mouse, α-SMA, Sigma A2547, 1:1000 dilution). A total of 5% skim milk in TBST was used for blocking followed by incubation with the appropriate secondary antibody. Proteins were visualized using a ECL Prime Western Blotting Detection Reagents (GE Healthcare, RPN2232). Blots were photographed with a Gel Doc machine (GelDoc Syngene GBox Chemi xrq), then analysis by densitometry of the blot was done using ImageJ software.

#### Statistical analysis

Continuous variables were associated by independent sample *t* test or ANOVA for normally distributed data, and Mann-Whitney test or Kolmogorov-Smirnov test for data thatwere not normally distributed. Values of *p* < 0.05 were considered statistically significant. Statistical analyses were accomplished using SPSS software version 17.0 (SPSS Inc., Chicago, IL, USA).

## Results

### Hyperuricemia induced glomerulosclerosis and tubular injury

**Fig. 1:**
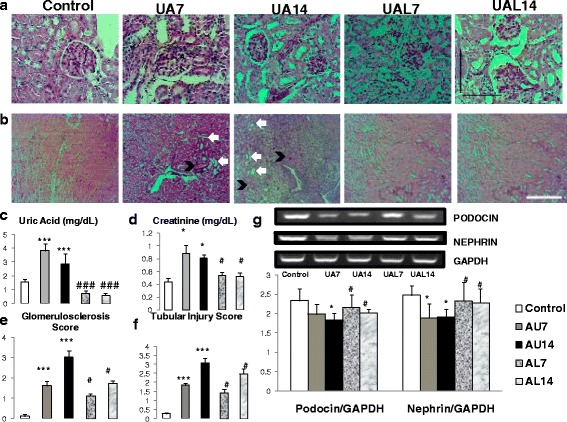
Uric acid treatment induced glomerulosclerosis and tubular injury with downregulation of podocyte’s markers. **a**-**b** Glomerulosclerosis representative picture based on PAS staining in 400× magnification, bar = 100 μm. **b** Tubular injury representative picture based on PAS staining in 100× magnification, bar = 100 μm. Tubular injury was characterized by tubular dilatation (white arrow) and intraluminal cast appearance (black arrow). Uric acid injection induced glomerulosclerosis and tubular injury in Swiss background mice. Reduction of uric acid level attenuated glomerulosclerosis and tubular injury. **c** Serum uric acid level is higher in UA7 and UA14 groups. **d**-**f** Quantification of creatinine level, glomerulosclerosis and tubular injury score in each group showed renal deterioration in UA group. **g** Down-regulation of nephrin and podocin mRNA expression in Reverse Transcripatase PCR (RT-PCR) showed podocyte injury in UA groups. *p < 0.05 VS control; ***p < 0.001 VS Control; #*p* < 0.05 VS UA14, ###*p* < 0.001 VS UA14

Uric acid injection induced hyperuricemia condition in mice as shown by a significant increase of serum uric acid level in day 7 and day 14 (Fig. 1). Increase of uric acid was associated with elevation of serum creatinine level which revealed deterioration of renal function. Allopurinol could reduce both serum uric acid and creatinine level to the normal level. Histopathology analysis revealed increased glomerulosclerosis and tubular injury in UA7 (*p* < 0.05 vs. Control) and UA14 (*p* < 0.05 vs. Control) groups. We also found a significant difference in the UA7 and UA14 groups (*p* < 0.05). The RT-PCR revealed reduction of nephrin and podocin expression in the hyperuricemia groups. Furthermore, reduction of uric acid level in the allopurinol group could ameliorate reduction of nephrin and podocin expression.

### Kidney inflammation and fibrosis occurred in hyperuricemia group

**Fig. 2:**
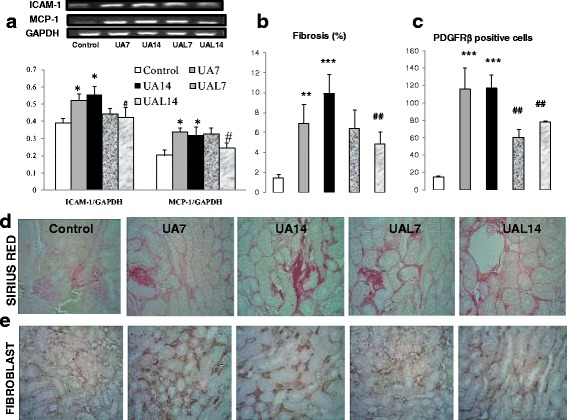
Uric acid treatment increased inflammation and fibrosis in kidney. **a-b** Reverse Transcriptase PCR showed inflammatory mediator (ICAM-1 and MCP-1) mRNA expression in UA group. **c-e** Sirius Red staining in 400× magnification and interstitial fibrosis area fraction quantification. It revealed higher quantification of area fraction in UA group and reduced in UAL7 and UAL14 groups. **d-e** Immunostaining of PDGFRβ as marker of fibroblast (400× magnification) to quantify fibroblast cell number. Fibroblast number/field was higher in UA group, and groups with alopurinol treatment (UAL7 and UAL14) had lower fibroblast number/field.*p < 0.05 VS control; ***p < 0.001 VS Control; #*p* < 0.05 VS UA14, ##*p* < 0.01 VS UA14

**Fig. 3:**
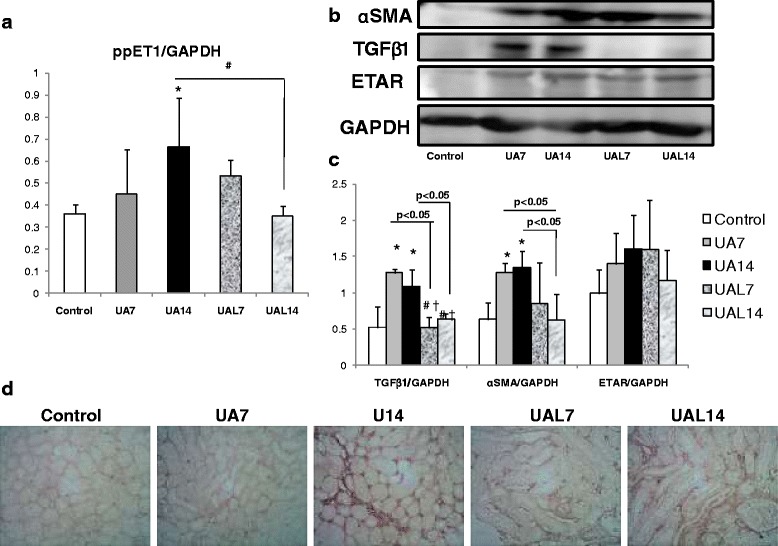
Uric acid treatment upregulated ET-1 mRNA, TGF-β1 and α-SMA protein expression with demonstrated myofibroblast formation. **a** Real Time PCR (qRT-PCR) quantification showed increase of ET-1 mRNA in UA group. **b**, **c** Westernblot representative picture and densitometry analysis revealed higher expression of TGFβ1 and αSMA protein expression in UA group. Allopurinol treatment induced downregulation of the protein expression especially in UAL14 group. **d** Immunohistochemical staining of αSMA (400× magnification) to examine positive staining in interstitial areas in UA14 group which represented some myofibroblast expansion. *p < 0.05 VS control group; #*p* < 0.05 VS UA14 group, † VS UA7 group

Inflammatory mediators’ expressions were up-regulated in UA7 and UA14 as shown by significant higher expression of ICAM-1 and MCP-1 compared to the Control group (Fig. 2a). Meanwhile, the allopurinol group especially UAL14 had significant lower expression of ICAM-1 and MCP-1. This finding might be associated with higher fibrosis area fraction in UA groups with higher fibroblast number (Fig. 2b-e). Quantification of collagen area fraction showed lower collagen area fraction between UA14 and UAL14 groups, with significant lower fibroblast cell number (Fig. 2c). Further analysis using qRT-PCR of ET-1 mRNA demonstrated significant higher expression of ET-1 mRNA between UA14 and Control groups. Furthermore, allopurinol effect might be seen in lower expression of ET-1 mRNA in the UAL14 group compared to UA14 (Fig. 3a). Immunoblot showed up-regulation of TGF-β1 and α-SMA protein expression in UA7 and UA14 groups. However, there were no significant differences in ETAR protein expression. Immunostaining of α-SMA as a myofibroblast marker represented positive staining in interstitial areas of the UA14 group. Reduction of positive staining can be seen in the UAL7 and UAL14 groups (Fig. 3d).

## Discussion

In this study we also found that hyperuricemia induced increased inflammation mediators such as MCP-1 and ICAM-1. Those effect might be mediated by reduction of NFκB, a nuclear transcription factor which plays a role in mediating inflammation. Renal epithelial cells have been known as a source of NFκB due to uric acid stimulation [21]. Reduction of uric acid level in the UAL7 and UAL14 groups produced reduction in this effect. This study confirmed Zhou’s et al.’s findings, which showed that induced hyperuricemia increased inflammatory mediators such as MCP-1, TNF-α and RANTES [22].

The process of uric acid in producing renal fibrosis and injury involves many mechanisms especially inflammation and fibroblast expansion. First we showed that 125 mg/kg body weight of daily uric acid injection intraperitoneally could produce hyperuricemia conditions after 7 days and 14 days. Uric acid also induced glomerulosclerosis and tubular injury, while a reduction of serum uric acid level reduced the glomerulosclerosis and tubular injury. Uric acid contributes to the development of end-stage renal diseases, especially before the availability of uric acid lowering agents [23]. Hyperuricemia also is recognized as an independent factor for progression of IgA nephropathy [24, 25]. Based on this study uric acid might become a predictor for the progression of renal diseases [26]. Epidemiological study also revealed that increased levels of uric acid to 8 mg/dL increases renal insufficiency to 2.9 times compared to uric acid with 5 mg/dL [27].

The effect of hyperuricemia in renal diseases might correlate with hypertension, intrarenal vascular disease, and renal injury [2]. Uric acid belongs to the Damage Associated Molucular Pattern (DAMP) and induces inflammation response. Interestingly, the study using transgenic uricase showed reduction of uric acid in interstitial fluid contributing to reduction of inflammation effect due to uric acid. It seems uric acid induces inflammation in the interstitial space [28]. Renal interstitial fluid has been shown to contribute to formation of myofibroblast cells which are responsible for matrix production during fibrosis. Pericytes and fibroblasts induce myofibroblast transition which then leads to kidney fibrosis. We used PDGFR-β to mark the renal interstitial cells. Many studies used the same antibody to examine fibroblasts [29] or pericytes [30].

One study had reported that uric acid induced secretion of fibronectin, a component of the extracellular matrix. This effect is mediated by URAT-1 Transporter [21]. Almost all urate is reabsorbed in the proximal tubules [31]. This process is facilitated by a specific transporter, involving a voltage-sensitive pathway and an urate/anion exchanger. One of these transporters is URAT-1 Transporter. Knockdown of URAT-1 Transporter in epithelial cells culture reduced uric acid induced-fibronectin secretion [21]. In this study we proposed the effect of uric acid in expansion of fibroblasts in-vivo. We found support that uric acid is transported through epithelial cells and acts in inducing fibronectin secretion [21]. Furthermore, we assumed this effect also might continue to interstitial areas consisting of many cells and structures, such as vessels, micro-vessels, fibroblasts and pericytes. Elevation of uric acid due to uricase inhibitor had been reported to induce thickening of afferent arteriole, renin-angiotensin system activation, and hypertension in rats [15, 16]. Uric acid also stimulates rat vascular smooth muscle cell (VSMC) proliferation in-vitro with increased expression of Platelet-Derived Growth Factor (PDGF), Cyclooxygenase-2, and Monocyte Chemoattractant Protein-1 [17, 18]. Interstitial fluid and space may contribute to the pathological effect of hyperuricemia. Uric acid might increase after cell death, then uric acid is secreted to extracellular fluids. High uric acid level may undergo nucleation due to high levels of sodium in extracellular fluids. This process induces inflammation related to DAMP [28]. Those effects might influence the biology of renal interstitial cells, such as fibroblasts. In this study we also revealed that fibroblasts underwent expansion in the UA7 and UA14 groups. We assumed that high levels of uric acid in interstitial spaces may contribute to the expansion of fibroblasts. Our immunoblot and q-RTPCR results showed higher expression of profibrotic factors such as TGF-β1 and ET-1 protein expression which confirm activation of fibrotic pathways after uric acid induction.

Uric acid not only induces endothelial dysfunction and vascular injury [32, 33], but also promotes CKDs through inducing epithelial to mesenchymal transition [34]. Many cells undergo mesenchymal transition thereby inducing the development of renal fibrosis, the final pathway of CKDs [29, 30, 35–37]. Uric acid might not only promote epithelial cells for mesenchymal transition, but also induce myofibroblast transition from fibroblast. Immunostaining of this study also demonstrated fibroblast expansion (Fig. 2e) and showed αSMA positive staining which represented myofibroblasts in interstitial areas of the UA groups (Fig. 3b-d). We proposed in this study that uric acid might induce renal injury through stimulation of fibroblast expansion to myofibroblast transition. Uric acid might induce fibrotic factors, such as TGF-β1 and ET-1, thereby inducing fibroblast expansion and myofibroblast formation. Further experiments using fibroblast culture with uric acid induction are needed to confirm our results.

We also found that high uric acid is associated with higher expression of ET-1 mRNA (Fig. 3a). We suggested that ET-1 may modulate effect of uric acid in glomerular injury. ET-1 also might be regulated by uric acid, then lead to proliferation of vascular smooth muscle cells of Human Vascular Smooth Muscle cells culture [38]. ET-1 might be secreted by cells in glomerulus and inducing glomerulosclerosis [39]. ET-1 also induces podocyte injury then lead to glomerulosclerosis [40]. Increased ET-1 mRNA had already been shown in congenital pelvic-ureteral junction obstruction in CKD patients [41]. Direct contribution of ET-1 in kidney fibrosis has been shown by studies using ET-1 transgenic mice. ET-1 transgenic mice underwent spontaneous glomerulosclerosis, interstitial fibrosis, and renal cysts but not hypertension [39]. Effects of uric acid in glomerular and tubulointerstitial injury might also be mediated by ET-1. Endothelin receptor-A blocker in the CKDs due to diabetes prevented cytoskeletal and foot processes effacement of podocyte. These effects were equal to Angiotensin Converting Enzyme Inhibitor, especially in reducing proteinuria and podocyte structural maintenance [1]. Although there are only a small number of studies on humans, the recent studies showed the cross-talk between ET-1 and Renin-Angiotensin System (RAS) to be the same as in the study in animals. Uric acid stimulates activation of RAS [32]. Goddard et al. showed that inhibition of endothelial receptor and ACE gives a synergistic effect in ameliorating renal blood flow and sodium excretion [42, 43]. In patients with CKDs due to proliferative glomerulonephritis, selective Endothelin A Receptor (ETAR) blocker could reduce blood pressure due to increasing vasodilation. This finding is associated with the role of ETAR in the efferent arteriole vasoconstriction effect [42]. However, we could not examine and determine significantly higher ETAR protein expression in the uric acid groups. Inflammation in the uric acid group might also be stimulated by higher ET-1 expression. Reduction of ET-1 in kidney ischemic reperfusion injury also showed reduction of inflammation with down-regulation of TLR-2, TLR-4, MCP-1, and ICAM-1 accompanied by reduction of macrophage number [44]. The contribution of the ET-1 pathway in uric acid induction still needs further investigation.

## Conclusion

In conclusion, this study highlighted the effect of uric acid in renal deterioration which might be mediated by induction of renal fibroblast expansion and ET-1 stimulation. Further research is needed to specifically elucidate the uric acid effect in renal fibroblast culture with ET-1 or ET receptor blocker addition.

## Abbreviations

DAMP: Damage Associated Molecular Pattern
ET-1: Endothelin-1
ETAR: Endothelin A receptor
ETBR: Endothelin B receptor
ICAM-1: Intercellular Adhesion Molecule-1
MCP-1: Monocyte Chemoattractant Protein-1
NFκB: Nuclear Factor Kappa B
PAS: Periodic Acid Schiff
PDGFRβ: Platelet Derived Growth Factor Receptor β
ppET-1: PreproEndothelin-1
RT-PCR: Reverse Transcriptase PCR
TGF-β1: Transforming Growth Factor-β1
TNF-α: Tumor Necroting Factor-α
VSMC: Vascular Smooth Muscle Cell
